# A new method for screening acute/chronic lymphocytic leukemia: dual-label time-resolved fluorescence immunoassay

**DOI:** 10.1186/s12896-022-00758-2

**Published:** 2022-09-30

**Authors:** Zhen Zhang, Jintao Zhang, Shanshan Dai, Hang Xu

**Affiliations:** 1 Department of Logistics Management, Baoshan Branch, Ren Ji Hospital, School of Medicine, Shanghai Jiao Tong University, Shanghai, 200444 China; 2 Department of Medical Intensive Care Unit, Baoshan Branch, Ren Ji Hospital, School of Medicine, Shanghai Jiao Tong University, Shanghai, 200444 China; 3 Department of Medical Engineering, Affiliated Cancer Hospital of Xinjiang Medical University, Xinjiang, 830000 China; 4 Department of Surgical Intensive Care Unit, Baoshan Branch, Ren Ji Hospital, School of Medicine, Shanghai Jiao Tong University, No.1058, Huan Zhen Bei Rd, Shanghai, 200444 China

**Keywords:** Time-resolved fluorescence immunoassay, Acute/chronic lymphocytic leukemia, Dual-label, S100A8, LRG1

## Abstract

**Background:**

Lymphocytic leukemia (LL) is a primary malignant tumor of hematopoietic tissue, which seriously affects the health of children and the elderly. The study aims to establish a new detection method for screening acute/chronic LL using time-resolved fluorescence immunoassay (TRFIA) via quantitative detection of S100 calcium binding protein A8 (S100A8) and leucine-rich alpha-2-glycoprotein 1 (LRG1) in serum.

**Methods:**

Here a sandwich TRFIA was optimized and established: Anti-S100A8/LRG1 caputre antibodies immobilized on 96-well plates captured S100A8/LRG1, and then banded together with the anti-S100A8/LRG1 detection antibodies labeled with Europium(III) (Eu^3+^)/samarium(III) (Sm^3+^) chelates. Finally time resolved fluorometry measured the fluorescence intensity.

**Results:**

The sensitivity of S100A8 was 1.15 ng/mL(LogY = 3.4027 + 0.4091 × LogX, *R*^*2*^ = 0.9828, *P* < 0.001, dynamic range: 2.1–10,000 ng/mL), and 3.2 ng/mL for LRG1 (LogY = 3.3009 + 0.4082 × LogX, *R*^*2*^ = 0.9748, *P* < 0.001, dynamic range: 4.0–10,000 ng/mL). The intra-assay and inter-assay CVs were low, ranging from 5.75% to 8.23% for S100A8 and 5.30% to 9.45% for LRG1 with high specificity and affinity in serum samples. Bland–Altman plots indicated TRFIA and ELISA kits have good agreement in clinical serum samples. Additionally, the cutoff values for S100A8 and LRG1 were 1849.18 ng/mL and 588.08 ng/mL, respectively.

**Conclusion:**

The present TRFIA method could be used for the quantitative detection of S100A8 and LRG1 in serum, and it has high sensitivity, accuracy and specificity. Clinically, this TRFIA method could be suitable for screening of LL via the quantitative detection of S100A8 and LRG1.

## Background

Lymphocytic leukemia (LL) is roughly divided into acute and chronic lymphocytic leukemia, and its lesions involve the surrounding blood, lymph nodes and organs throughout the body [1]. Acute LL occurs in both children and adults, and its incidence peaks between 2 and 5 years old, while chronic LL occurs in middle-aged and elderly patients [2]. At present, the treatment of LL remains a challenging clinical issue despite remarkable improvements in prognostication and therapy [3]. Therefore, early screening is especially important in the treatment of LL, specific biomarkers testing is the new method for LL screening.

S100 calcium binding protein A8 (S100A8) is a member of the S100 calcium-binding protein family, it associated with myeloid cell differentiation/apoptosis, adhesion of neutrophils, and chemotactic activities etc. [4, 5]. The medical community has increasingly recognized that S100A8 is one of the biomarkers of malignant tumors [4–6]. Recent studies revealed that S100A8 plays an important role in the apoptosis/proliferation of leukemia cells [4]. S100A8 expression in leukemic cells could predict survival in acute myeloid leukemia patients, and the patient with high S100A8 level had the low overall survival [7]. In drug resistance leukemia cells, chemotherapy agents could induce S100A8 expression and S100A8 elevated [8]. Proteomics research found that S100A8 were associated with high-risk CLL, and the high S100A8 expression may be associated with the poor prognosis, indicating S100A8 may be a new marker for individualized precise treatment of children with ALL [9, 10]. So far, ELISA is the most commonly used method for detecting serum S100A8 levels in clinical practice [11, 12].

Leucine-rich alpha-2-glycoprotein-1 (LRG1) is a serum glycoprotein and have been increasingly recognized as biomarkers for certain diseases including microbial infections and cancer [13]. LRG1 was found to be significantly elevated in the sera of some patients with colorectal cancer and bacterial infection [14, 15]. LRG1 regulates tumor proliferation and apoptosis in acute myeloid leukemia (AML) cell lines, LRG1 expression increased in AML cells lines and LRG1 gene silencing could reduce cell viability and promote apoptosis [16]. Xiao et al. found the expression levels of LRG1 significantly increased in hematological malignancies, which is related to the pathogenesis of childhood hematological malignancies [17]. The existing clinical detection method of LRG1 is mainly ELISA, but also include two-dimensional liquid chromatography and tandem mass spectrometry and nanosensors [17, 18].

Based on the above information, we hypothesized that S100A8 and LRG1 are specific biomarkers for acute/chronic LL. Therefore, we aimed to establish a new method to screen acute/chronic LL via the quantitative detection of S100A8 and LRG1. Time-resolved fluorescence immunoassay (TRFIA) is a promising detection technique, and has been widely used in various fields, including antigen, antibody and viruses detection [19, 20]. In this study, we have optimized and established a TRFIA method for S100A8 and LRG1 quantitative detection.

## Results

### Assay optimization

**Fig. 1:**
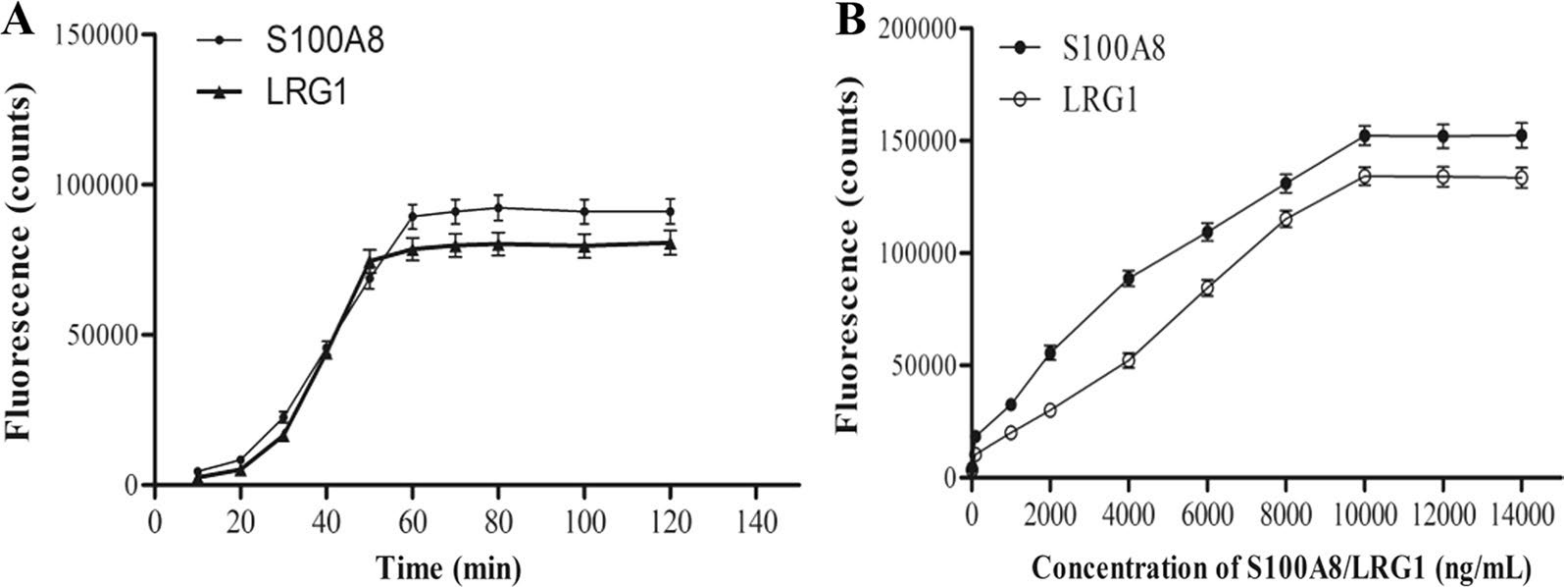
Optimization of immunoreaction time (**A**) and hook effect (**B**)

The final optimization conditions are as follows: coating concentration of S100A8: 3 μg/mL, 100 μL/well; coating concentration of LRG1: 2 μg/mL, 100 μL/well; the optimal ratio of Eu^3+^ labels and S100A8 detection antibody: 100 μg:1 mg, and the optimal ratio of Sm^3+^ labels and LRG1 detection antibody: 150 μg:1 mg; the sample volume: 30 μL/well; Eu^3+^/Sm^3+^ labeled antibodies volume: 100 μL/well Eu^3+^-labeled antibody + 100 μL/well Sm^3+^-labeled antibody; enhancement solution volume: 200 μL/well, and the immunoreaction time was 1 h (Fig. 1A). Therefore, the optimized assay procedure is: 30 μL serum samples or standards, 100 μL Eu^3+^-labeled S100A8 detection antibody and 100 μL Sm^3+^-labeled LRG1 detection antibody were added into the coated 96-well plates, and then incubated for 60 min at room temperature. After washed the wells, added 200 μL/well enhancement solution into the wells, and shaken gently for 2 min. Finally, time-resolved analyzer measured the fluorescence.

### TRFIA standard curves

**Fig. 2:**
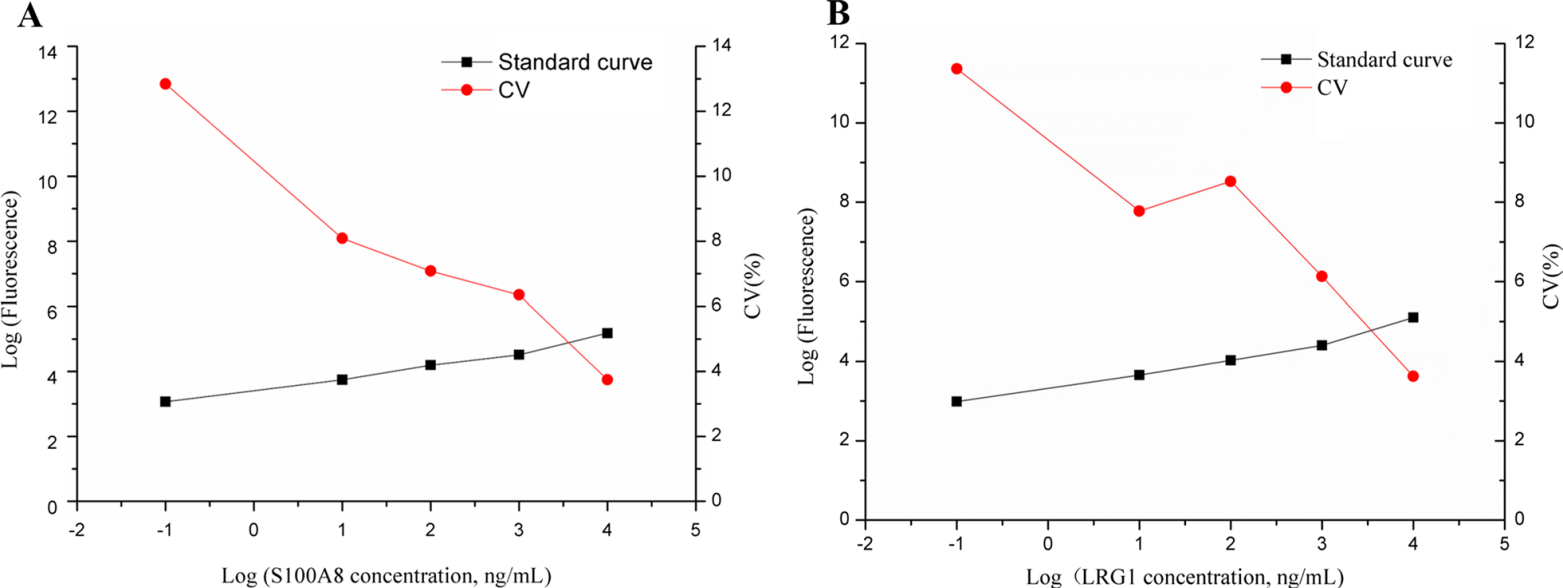
The standard curves of S100A8 (**A**) and LRG1 (**B**). Standard curve determinations were carried out using linear regression and log–log regression. The Log function values of S100A8/LRG1 standards were plotted as X axis, and the Log function value of their fluorescence as the Y axis, performed a linear fit and draw a standard curve

Hook effect was seen when the range exceeded 10,000 ng/mL (Fig. 1B). Standard curve determinations were carried out using linear regression and log–log regression. For the standard curve depicted in Fig. 2, the best-fit calibration was determined to be described by the following equation: S100A8: LogY = 3.4027 + 0.4091 × LogX (*R*^*2*^ = 0.9828, *P* < 0.001), LRG1: LogY = 3.3009 + 0.4082 × LogX (*R*^*2*^ = 0.9748, *P* < 0.001). Graphical estimation indicates the LOD of S100A8 was 1.15 ng/mL(dynamic range: 2.1–10,000 ng/mL), and LOD of LRG1 was 3.2 ng/mL (dynamic range: 4.0–10,000 ng/mL).

### Specificity results

The present TRFIA detected the concentrations of five interferents (TNF-α, CEA, TRF, hs-CRP and HSA) with high concentrations, and the results are shown in Table 1. Table 1 indicated that there was only very low cross-reactivity, and the present TRFIA method had high specificity and affinity for S100A8 and LRG1.

**Table 1:** Specificity of the dual-label TRFIA

Interferents	Nominal concentration (ng/mL)	Mean ± SD (ng/mL)	Cross-reactivity (%)
S100A8	2000	1945.25 ± 135.64	97.26
LRG1	2000	1932.25 ± 187.42	96.61
TNF-α	2000	3.54 ± 1.65	0.68
CEA	2000	5.21 ± 1.24	0.76
TRF	2000	5.20 ± 1.17	0.76
hs-CRP	2000	4.26 ± 1.39	1.21
HSA	2000	6.27 ± 1.35	0.81

### Accuracy and recovery results

As presented in Table 2, the intra-assay and inter-assay CVs were low, ranging from 5.75% to 8.23% for S100A8 and 5.30% to 9.45% for LRG1. All CVs were less than 10%, which indicates that this TRFIA assay has high accuracy. Additionally, the recovery of S100A8 was between 92.50% and 106.54%, while that of LRG1 was between 90.75% and 105.64%. The recovery results indicated that this TRFIA method was free from interferences in serum samples.

**Table 2:** Accuracy and recovery of the dual-label TRFIA

	S100A8			LRG1		
Theoretical (ng/mL)	Mean ± SD (ng/mL)	CV(%)	Recovery (%)	Mean ± SD (ng/mL)	CV(%)	Recovery (%)
*Inter-assay (n* = *10)*						
100	106.54	7.64	106.54	105.64	9.45	105.64
2000	1948.25	8.23	97.41	1905.58	7.98	95.28
5000	4857.48	6.25	97.15	4618.67	5.93	92.37
*Intra-assay (n* = *10)*						
100	92.50	6.82	92.50	90.75	8.24	90.75
2000	2105.49	7.26	105.27	2085.62	6.83	104.28
5000	5124.6	5.75	102.49	5241.06	5.30	104.82

## Comparison results

**Fig. 3:**
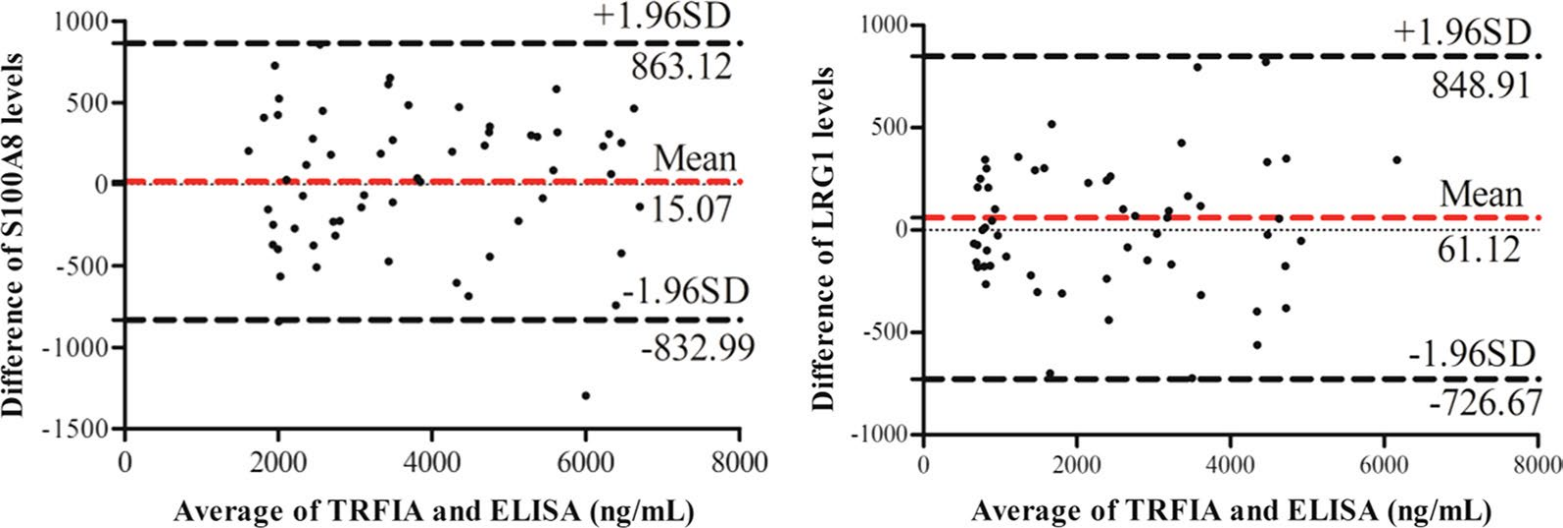
Bland–Altman plots of S100A8/LRG1 levels measured by TRFIA and ELISA kits

The comparison results are shown in Fig. 3. For S100A8, 95% limits of agreement was from − 832.99 ng/mL to 863.12 ng/mL, 1/59 of the points were outside this limit, and the average of the two methods was 15.07 ng/mL. For LRG1, 95% limits of agreement was from − 726.67 ng/mL to 848.91 ng/mL, none point was outside this limit, and the average of the two methods was 61.12 ng/mL. The results indicated that the TRFIA and the ELISA kits have good agreement in clinical serum samples.

### Reference intervals

**Fig. 4:**
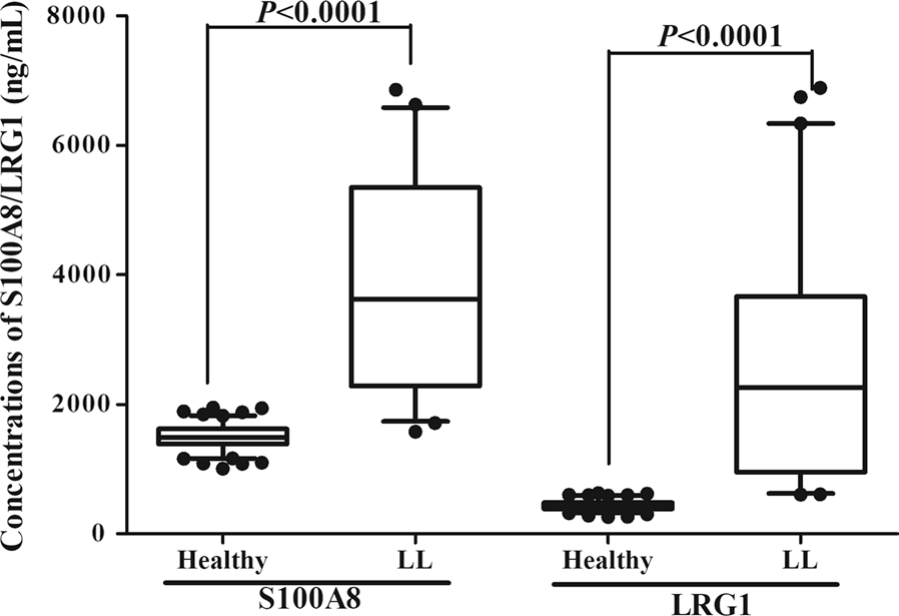
Test results for 179 serum samples, including 120 healthy serum samples and 59 serum samples with LL

For 120 clinically healthy serum samples, the mean S100A8 concentration was 1499.48 ng/mL, the SD was 178.42, while the mean LRG1 concentration was 437.83 ng/mL, and the SD was 76.66, so the cutoff value of S100A8 was 1849.18 ng/mL and 588.08 ng/mL for LRG1, which meant that when the concentration of S100A8 was higher than 1849.18 ng/mL and the LRG1 concentration was higher than 588.08 ng/mL, the provider of this serum sample may be suffering from lymphocytc leukemia. Additionally, statistical analysis of the test results of 120 healthy serum samples and 59 serum samples with LL found that the concentrations of S100A8 and LRG1 in the lymphocytc leukemia patients was significantly higher than that of healthy human (*P* < 0.001, Fig. 4), indicating that this TRFIA method may be used for LL screening.

## Discussion

For the screening and diagnosis of LL, conventional genetic testing, flow cytometry testing and hematological testing have been commonly used in clinical practice, but these methods have expensive and time-consuming. Recently, a variety of specific biomarkers have been discovered and used in the clinical screening and diagnosis of LL [21, 22]. Due to the high sensitivity and specificity, dual-marker and multi-marker testing is necessary for clinical practice. For example, the simultaneous determinaton of ferritin and β2‐microglobulin could be used for the early screening and follow-up surveillance of the acute and chronic LL [23]. Comparison results of the concentrations of S100A8 and LRG1 in 120 healthy serum samples and 59 serum samples with LL, the concentrations of S100A8 and LRG1 in the LL patients was significantly higher than that of healthy human. Based on the findings of S100A8/LRG1 levels in previous reports and our study [4–18], we inferred that S100A8 and LRG1 may be the specific markers for LL, and the quantitative detection of S100A8 and LRG1 in serum could be suitable for screening of LL. Therefore, we established a new method for screening acute/chronic lymphocytic leukemia using TRFIA.

TRFIA is a simple, sensitive, fast and cost-effective fluoroimmunoassay. Eu^3+^ fluorescence was measured at an excitation wavelength of 337 nm and an emission wavelength of 615 nm, and Sm^3+^ fluorescence was measured at an excitation wavelength of 340 nm and an emission wavelength of 600 nm [24]. Such obvious wavelength gap makes TRFIA free of nonspecific background, and suitable for various samples detection. Actually, TRFIA has been used for the detection of antigen, antibody and viruses in various body fluids such as serum, plasma and urine [19, 20, 25]. Importantly, in previous study, TRFIA has been used for the early screening of the acute and chronic LL. Liu et al. reported that dual-label TRFIA had been developed for the determinaton of ferritin and β2‐microglobulin, which may be used for the early screening and follow-up surveillance of the acute and chronic LL [23]. Therefore, the present TRFIA method is feasible for LL screening via the quantitative detection of S100A8 and LRG1 in serum.

In this study, we optimized and established a sandwich TRFIA method with high sensitivity, specificity, and accuracy for the quantitative detection of S100A8 and LRG1 in serum. The sensitivity of S100A8 was 1.15 ng/mL, and 3.2 ng/mL for LRG1. The intra-assay and inter-assay CVs were low with high specificity and affinity in serum samples. There was a high *Pearson* coefficient between the present TRFIA method and a commercially ELISA kit. Additionally, we calculated the cutoff values of S100A8 and LRG1 were 1849.18 ng/mL and 588.08 ng/mL respectively using 120 healthy serum samples. That means when the concentration of S100A8 was higher than 1849.18 ng/mL and the LRG1 concentration was higher than 588.08 ng/mL, the provider of this serum sample may be suffering from lymphocytc leukemia. Compared with the S100A8 and LRG1 ELISA kits, the TRFIA has some superiority. The whole assay procedure of ELISA kit requires more than 4 h (primary antibody incubation for 2.5 h, secondary antibody incubation for 1 h, color reaction for 30 min, and wash time etc.), while this TRFIA only needs approximately 70 min (incubation time 60 min, a few minutes of wash time and excitation time), which will save approximately 3 h in detection time. Additionally, this TRFIA kit can detect two indicators (S100A8 and LRG1) at once, simultaneous detection of multiple indicators can more accurately assist the diagnosis results, reduce diagnosis time and save money. Moreover, the given cutoff values and reference intervals of S100A8/LRG1 can facilitate the assessment of the patient's physical condition. Although the sensitivity of TRFIA is not as good as that of ELISA, it is because TRFIA is designed according to the actual level of S100A8/LRG1 in clinical serum samples, so that the obtained serum samples can be directly used for detection without dilution, which simplifies the detection steps.

## Conclusion

In conclusion, a dual-label TRFIA with high sensitivity, specificity, and accuracy was developed for the quantitative detection of S100A8 and LRG1 in serum. Clinically, this TRFIA method could be suitable for screening of LL via the quantitative detection of S100A8 and LRG1. However, although we have established a dual-label TRFIA detection method for S100A8 and LRG1 in serum, its clinical application still needs more clinical samples verification, and other clinical diagnostic methods are needed to confirm the diagnosis of LL.

## Materials and methods

### Antigen, antibody and samples

The S100A8 antigen, LRG1 antigen, and their capture and detection antibodies were purchased from Yidenuo Bio-technology (Guangzhou, China). 59 clinical serum samples with acute/chronic LL and 120 healthy serum samples came from the Baoshan Branch of Renji Hospital Affiliated to Shanghai Jiaotong University, and all serum samples were stored at − 80 °C. All participants or their guardians gave written informed consent, and this study was approved by the Institutional Review Board of Baoshan Branch of Renji Hospital Affiliated to Shanghai Jiaotong University.

### Reagents, instrumentation and solutions

Europium(III) (Eu^3+^) and samarium (III) (Sm^3+^) labels were purchased from PerkinElmer (Norwalk, USA). Tween-20, bovine serum albumin (BSA), triton X-100, β-naphthoyltrifluoroacetate and tri-n-octylphosphine oxide were procured from Sigma-Aldrich (St.Louis, USA). Other chemicals and reagents used were analytical grade. Sephadex G50 column was purchased from GE Healthcare Life Sciences (Marlborough, MA, USA). 96-well plates and time-resolved analyzer (Auto 1420) were purchased from PerkinElmer (Waltham, MA, USA). Washing buffer: 20 mmol/L Tris-HCl (pH 8.0) containing 0.05% Tween-20 and 0.9% NaCl. Blocking buffer: 50 mmol/L PBS (pH 7.4) containing 4% BSA. Labeling buffer: 50 mmol/L Na_2_HCO_3_-Na_2_CO_3_ (pH 9.0) containing 0.9% NaCl. Assay bufer: 50 mM Tris-HCl (pH 7.8) supplemented with 0.05% Tween-20 and 0.02% Proclin300. Enhancement solution: 0.1 mol/L acetate-phthalate buffer (pH 3.2) supplemented with 0.1% Triton X-100, 30 μmol/L β-naphthoyltrifluoroacetate, 60 μmol/L tri-n-octylphosphine oxide and 0.5% glacial acetic acid.

### Detection antibody labeling

Eu^3+^ and Sm^3+^ labels were used for S100A8 and LRG1 detection antibody labeling. Optimized the ratio of Eu^3+^/Sm^3+^ labels and detection antibodies. Briefly, the detection antibodies of S100A8 were dissolved in labeling buffer, and then added the Eu^3+^ chelates. The mixture was gently shaken and incubated for 24 h at 4 °C, and then purified the Eu^3+^-labeled S100A8 detection antibodies using the Sephadex G50 column. Finally, the Eu^3+^-labeled S100A8 detection antibodies were stored at 4 °C in the dark. The same procedure was used for Sm^3+^-labeled LRG1 detection antibody labeling.

### Capture antibody coating

S100A8 capture antibody and LRG1 capture antibody were simultaneously coated into 96-well plates. Prepared a various of S100A8/LRG1 capture antibodies concentrations to optimize the S100A8/LRG1 capture antibodies coating concentration. Briefly, added the S100A8/LRG1 capture antibodies into the 96-well and incubated for 2 h at 37 °C. After washed three times, the plates were blocked with 200 μL/well blocking buffer for 1 h at 37 °C. Then, removed the blocking buffer and dried in vacuum, and finally stored at − 20 °C.

### Assay procedure

**Fig. 5:**
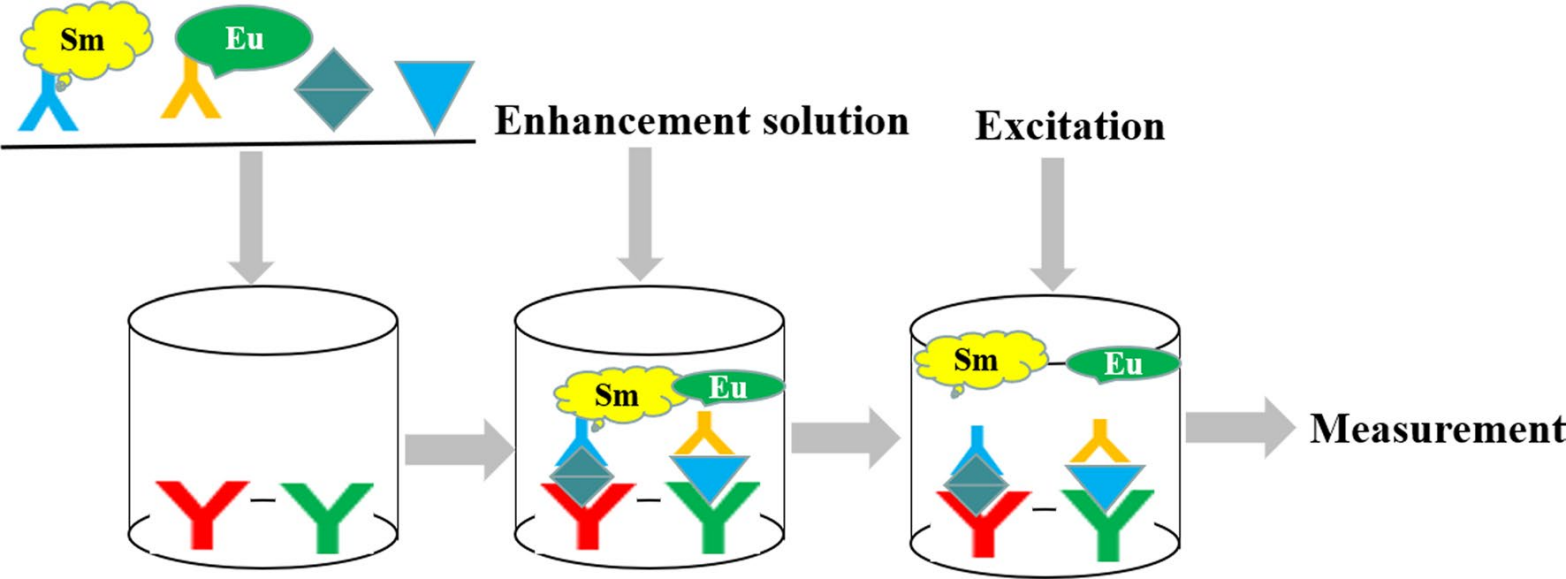
Scheme of the dual-label TRFIA for S100A8 and LRG1 detection

Optimized assay procedure using the one-step procedure, including the sample volume, Eu^3+^/Sm^3+^ labeled antibodies volume, enhancement solution volume, and the total reaction time. All the assay procedure were set in advance and preformed the automatic detection by the time-resolved analyzer (Auto 1420, PerkinElmer). Briefly, serum sample, standard or controls, and Eu^3+^/Sm^3+^ labeled detection antibodies were added into the 96-well plates, and then incubated the plates at room temperature. After washed the wells three times, added the enhancement solution into the wells, and shaken gently for another 5 min. Finally, time-resolved analyzer measured the fluorescence. The scheme of the dual-label TRFIA for S100A8 and LRG1 is shown in Fig. 5. The optimized TRFIA method detected the fluorescence of the 2000 ng/mL S100A8/LRG1 standards at 10, 20, 30, 40, 50, 60, 70, 80, 100 and 120 min incubation time, respectively, to obtain the optimal immunoreaction time. The optimized TRFIA method detected the fluorescence of the serial dilutions of S100A8/LRG1 standards (0, 2000, 4000, 6000, 8000, 10,000, 12,000 and 14,000 ng/mL), to check the hook effect of this method.

### Standard curves and sensitivity assay

The standard curve was established using serial dilutions of S100A8 (0, 0.1, 1, 10, 100, 1000 and 10,000 ng/mL) and LRG1 (0, 0.1, 1, 10, 100, 1000 and 10,000 ng/mL) protein. The limit of the blank (LOB) was defined as the mean of the blank assayed in 20 independent measurements. The limit of detection (LOD) was determined by adding two standard deviations (SD) to the LOB, that means LOD = LOB + 2 *SD [26].

### Specificity assay

High concentrations of tumor necrosis factor-α (TNF-α), high sensitivity C-reactive protein (hs-CRP), carcino-embryonic antgen (CEA), Human transferrin (TRF), and human serum albumin (HSA) were selected for specificity assay. Data were obtained from eight independent experiments. High concentrations of interferences mentioned above were added into the healthy control serum and detected by this TRFIA. TNF-α and hs-CRP are the common and highly expressed inflammatory factors in the LL patients’ serum. CEA and TRF are the most commonly used tumor marker with highly expression in the LL patients’ serum. HSA was used to evaluate whether the serum matrix has an effect on the specificity of this assay.

### Accuracy and recovery assays

By adding different concentrations of S100A8 and LRG1 standards into the serum samples, we evaluated the accuracy and recovery of this method. Ten independent experiments were performed, and the intra-assay and interassay variations were obtained. The formula of recovery is: Recovery (%) = (measured concentration/theoretical concentration) × 100.

### Comparison assay

59 clinical serum samples with acute/chronic LL were simultaneously measured by this TRFIA method, human S100A8 ELISA kit (Sigma-Aldrich, #RAB0730, USA) and LRG1 ELISA kit (CUSABIO, #CSB-E12962h, China). Bland–Altman plots were used to compare the consistency of the TRFIA and the ELISA kits.

### Reference intervals

Serum samples from 120 healthy human was used to identify the reference intervals. The S100A8 and LRG1 concentrations of the 120 healthy human were detected by this dual-label TRFIA method. The S100A8 and LRG1 concentrations were performed a normality test with SPSS 17.0 respectively, and applied the one-sided upper limit of the 95% reference interval range to identify the reference intervals. Therefore, the cutoff values were calculated using the following formula: cutoff value = mean + 1.96SD [27].

### Statistical analysis

Data were statistically analyzed using SPSS 17.0, and graphed using GraphPad Prism 5 (GraphPad Software, USA) and Origin 8.0 (OriginLab, USA). The data are expressed as the mean ± SD. Correlations among different methods were calculated with Pearson correlation.

## Data Availability

All data generated or analysed during this study are included in this published article.

## Abbreviations

LL: Lymphocytic leukemia
RFIA: Time-resolved fluorescence immunoassay
S100A8: S100 calcium binding protein A8
LRG1: Leucine-rich alpha-2-glycoprotein 1
Eu^3+^: Europium (III)
Sm^3+^: Samarium (III)
LOB: Limit of the blank
LOD: Limit of detection
SD: Standard deviations
TNF-α: Tumor necrosis factor-α
hs-CRP: High sensitivity C-reactive protein
CEA: Carcino-embryonic antgen
TRF: Human transferrin
HSA: Human serum albumin
